# Efficient compressed database of equilibrated configurations of ring-linear polymer blends for MD simulations

**DOI:** 10.1038/s41597-022-01138-3

**Published:** 2022-02-08

**Authors:** Katsumi Hagita, Takahiro Murashima, Masao Ogino, Manabu Omiya, Kenji Ono, Tetsuo Deguchi, Hiroshi Jinnai, Toshihiro Kawakatsu

**Affiliations:** 1 Department of Applied Physics, National Defense Academy, 1-10-20, Hashirimizu, Yokosuka, 239-8686 Japan; 2 Department of Physics, Tohoku University, 6-3, Aramaki-aza-Aoba, Aoba-ku, Sendai, 980-8578 Japan; 3 Faculty of Informatics, Daido University, 10-3 Takiharu-cho, Minami-ku, Nagoya, 457-8530 Japan; 4 Information Initiative Center, Hokkaido University, Kita 11, Nishi 5, Kita-ku, Sapporo, 060-0811 Japan; 5 Research Institute for Information Technology, Kyushu University, 744 Motooka, Nishi-ku, Fukuoka, 819-0395 Japan; 6 Department of Physics, Ochanomizu University, 2-1-1 Ohtsuka, Bunkyo-ku, Tokyo, 112–8610 Japan; 7 Institute of Multidisciplinary for Advanced Materials, Tohoku University, 2-1-1 Katahira, Aoba-ku, Sendai, 980-8577 Japan

**Keywords:** Polymers, Nanocomposites, Applied mathematics, Statistical physics

## Abstract

To effectively archive configuration data during molecular dynamics (MD) simulations of polymer systems, we present an efficient compression method with good numerical accuracy that preserves the topology of ring-linear polymer blends. To compress the fraction of floating-point data, we used the Jointed Hierarchical Precision Compression Number - Data Format (JHPCN-DF) method to apply zero padding for the tailing fraction bits, which did not affect the numerical accuracy, then compressed the data with Huffman coding. We also provided a dataset of well-equilibrated configurations of MD simulations for ring-linear polymer blends with various lengths of linear and ring polymers, including ring complexes composed of multiple rings such as polycatenane. We executed 10^9^ MD steps to obtain 150 equilibrated configurations. The combination of JHPCN-DF and SZ compression achieved the best compression ratio for all cases. Therefore, the proposed method enables efficient archiving of MD trajectories. Moreover, the publicly available dataset of ring-linear polymer blends can be employed for studies of mathematical methods, including topology analysis and data compression, as well as MD simulations.

## Abstract (summary)

**Table:** 

Measurement(s)	equilibrated configurations of ring-linear polymer blends
Technology Type(s)	molecular dynamics simulation
Factor Type(s)	length of linear and ring polymer

Machine-accessible metadata file describing the reported data: 10.6084/m9.figshare.18742097

## Background & Summary

Molecular dynamics (MD) simulations are powerful tools for elucidating molecular-level behavior not only in biomolecular systems but also in polymer material sciences^1–4^. In MD simulations, coordinate data are recorded for detailed analyses. For such analyses, it is necessary to develop mathematical methods that can accurately evaluate how the linear chain penetrates the ring polymer; this has long been an important problem in the mathematics of topology^5–14^. The relevance of this task is not limited to ring-linear polymer blends^13,14^; research on knots in proteins^15–18^, threading of ring polymers^19–22^, and cross-linked networks^23,24^ is greatly concerned with the linkage between loops and chains owing to its impact on the material properties. Therefore, public availability of MD coordinate data is expected to promote the development of analysis methods by applied mathematicians.

Recently, there has been increasing attention in the field of polymer materials on mixed systems of ring and linear polymers. This is because recent experimental results have demonstrated the toughness of cross-linked ring-linear polymer blends^25,26^. Here, ring polymers work as movable cross-linking points to prevent stress concentration^25,26^. To understand these systems, it is important to first conduct detailed investigations of the equilibrium states of the ring-linear polymer blends. The equilibrium state can be obtained by long-term MD simulations^13,14^ in systems with a large number of ring and linear polymers; however, this is not an easy task. Thus, it is desirable to improve global efficiency through data sharing and reuse instead of duplicating calculations for multiple groups.

A mechanism for the efficient sharing data with reduced data sizes is important because datasets of MD trajectory data are typically very large. Moreover, compression of floating-point data is a common problem for scientific simulations in high-performance computing (HPC)^27–36^. Some studies on data compression^27–29^ found that the tailing fraction bits are too random to effectively compress because the tail bits in the fraction part of floating-point values in scientific data are more random than the head bits. Methods to neglect tail bits include error-controlled lossy data-compression methods such as ZFP^30^, ISABELA^31^, SSEM^32^, and SZ^33–36^. Recently, comparisons of compressor performance have been performed using benchmark data in various scientific domains; for example, for ZFP and SZ by Lu *et al*.^37^, Tao *et al*.^38^, and Cappello *et al*.^39^. As a result, SZ is regarded as a standard efficient compressor in HPC research for exascale computing. Note that Di and Cappello^40^ reported that time-trajectory analysis-based compressors^41–48^ become impractical in extremely large-scale particle simulations owing to their limited memory capacity. Thus, we focus on the data compression of snapshots.

For lossy compression of MD trajectory data in polymer systems, the required numerical accuracy (error level) and physical meanings such as preservation of topology should remain unchanged. Moreover, in the bit string of the coordinate data in polymer systems, the bits in the sequence along a chain have similar characteristics to time-series data in scientific simulations. Several authors^29,49,50^ have proposed the Jointed Hierarchical Precision Compression Number - Data Format (JHPCN-DF) method, which is a hierarchical segmented recording based on the required numerical precision (error level).

In this study, we analyze the relationship between the numerical accuracy and topology preservation of polymer MD trajectory data under JHPCN-DF compression with the aim of developing a publicly available database. The examined datasets consist of multiple melt systems with a mixture of ring polymers and linear chains. These datasets were prepared as well-equilibrated initial configurations for subsequent MD simulations in order to measure the rheological^51^ and mechanical properties after setting crosslinks. Note that these shared dataset provided the first successful discovery^51^ of a viscosity overshoot under biaxial extensional flows. In addition, these datasets are appropriate for the development of more accurate and rigorous mathematical judgment methods^52^, as well as efficient approximation techniques based on primitive path (PP) analysis^53^. As these datasets provide equilibrium states, they can also be useful for developing further coarse-grained MD models that reproduce these states^54^ and planning neutron scattering experiments to observe ring shapes in ring-linear blends. Moreover, publicly available data of polymer systems can be used as a benchmark dataset in the data-compression research community.

## Method

### Molecular dynamics simulations of ring-linear polymer blends

**Fig. 1:**
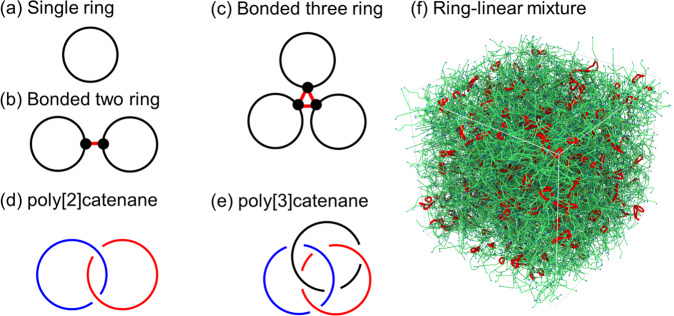
Schematics of single ring, bonded-rings, poly-catenanes, and ring-linear mixture. The snapshot of the ring-linear mixture with primitive path (PP)^53^ presentations for *N*_ring_ = *N*_linear_ = 160 with ring fraction 0.1 was rendered by OVITO^60^. In (**f**), ring polymers and linear chains are shown in red and green, respectively. The ends of linear chains are shown in blue.

We generated a dataset that included all combinations of the parameter conditions shown in Table 1 by performing MD simulations^13,14^. In all cases, MD simulations with a long length of 10^9^ MD steps were performed to obtain a well-equilibrated configuration of ring-linear polymer blends. Figure 1 presents schematics of the ring complexes. The examined system size was approximately 600,000 beads. The box sizes of the periodic boundary condition (PBC) were approximately (80)^3^ in the scale units. Note that the numbers of ring polymers and linear chains were included in the filename for each binary file.

**Table 1:** Parameter conditions.

Item	Values
Type of ring complex	single, bonded-two-ring, bonded-three-ring, poly [2]catenane, poly [3]catenane
Number of beads in a ring polymer (*N*_ring_)	80, 120, 160
Number of beads in a linear chain (*N*_linear_)	10, 20, 40, 80, 160
Ring fraction	0.05, 0.1

To obtain equilibrated configurations of ring-linear polymer blends, we performed coarse-grained MD simulations of the Kremer-Grest model^55^. Ring polymers with bead number *N*_ring_ and linear chains with length *N*_linear_ were placed in a box with PBCs, where the numbers of ring and linear polymers were *M*_ring_ and *M*_linear_, respectively. The length of each simulation run was 10^9^ MD steps with a time step (Δ*t*) of 0.005*τ*, where *τ* is a time unit.

In the KG model, the Lennard–Jones (LJ) potential with a cutoff length of *r*_c_ was applied to every pair of particles.

$${U}_{{\rm{LJ}}}(r)=4\varepsilon \left[{\left(\frac{\sigma }{r}\right)}^{12}-{\left(\frac{\sigma }{r}\right)}^{6}-{\left(\frac{\sigma }{{r}_{{\rm{c}}}}\right)}^{12}+{\left(\frac{\sigma }{{r}_{{\rm{c}}}}\right)}^{6}\right]$$

when *r* < *r*_c_, whereas *U*_LJ_(*r*) = 0 when *r* ≥ *r*_c_, where *r* is the distance between the beads, *ε* is the interaction strength, *σ* is the scale unit, and *r*_c_ is the cutoff length of the interaction. For simplicity, we set *ε* =  *σ* = 1 hereafter. To reproduce the excluded volume of chains with minimal computing costs, we set *r*_c_ to 2^1/6^. For bonded beads, the finite extensible nonlinear elastic (FENE) potential was also applied, where

$${U}_{{\rm{F}}{\rm{E}}{\rm{N}}{\rm{E}}}(r)=-\,\frac{k}{2}{R}_{0}^{2}\,{\rm{l}}{\rm{n}}\left[1-{\left(\frac{r}{{R}_{0}}\right)}^{2}\right]$$

for *r* < *R*_0_ and *U*_FENE_(*r*) = 0 for *r* ≥ *R*_0_. Here, *k* is the spring constant and *R*_0_ is the maximum bond length. The LJ and FENE potentials with *k* = 30 and *R*_0_ = 1.5 are widely used to prevent chains from crossing each other. The ring and linear polymers were placed in a box under PBCs with a bead number density of 0.85. Additionally, all ring polymers were unconcatenated. The bead dynamics in our model were described by a Langevin equation with a friction constant () of 0.5 *mτ*^−1^ and a temperature *T*. For simplicity, we set the mass of a bead (*m*) to unity so that *T* and LJ time (*τ* = *σ*(*m*/*ε*)^1/2^) became unity. The velocity Verlet algorithm was used for numerical integration of the Langevin equation. In this study, we used LAMMPS^56^ and HOOMD-blue^57^ MD simulation software.

### Topology judgement method of chain-penetration into a ring

We evaluated the Gauss Linking Numbers (GLNs) for all ring–linear pairs. However, GLNs cannot be applied to a ring and a linear chain unless the latter is a closed loop. In practice, the ends of linear chains are virtually connected to each other, but we prepared an extra linear chain and connected it to the original linear chain to form a cyclic chain. Details of this method were given in our previous work^13,14^. To compute GLNs among cyclic chains and ring polymers, we used the Topoly Python package^58^. For a catenated cyclic chain and ring pair, the GLN was equal to 1. Otherwise, GLN = 0. When GLN = 1, we concluded that the linear chain had penetrated the target ring chain.

### Efficient compression of floating data

To achieve efficient sharing of lossy and lossless compressed data, the JHPCN-DF method^29,49,50^ was used for hierarchical segmented recording based on the required numerical precision (error level). In essence, the JHPCN-DF framework involves lossless compression with segmented recording; for users who employ parts of the recording, it works as lossy compression. One of the merits of this framework is a substantial reduction of data transfer from big supercomputers to front-end computers for data confirmation through visualization. It should be noted that the part of compression related to the first fraction bits can be regarded as the same as masked data compression^28^, which was proposed independently by Gomez and Cappello.

**Fig. 2:**
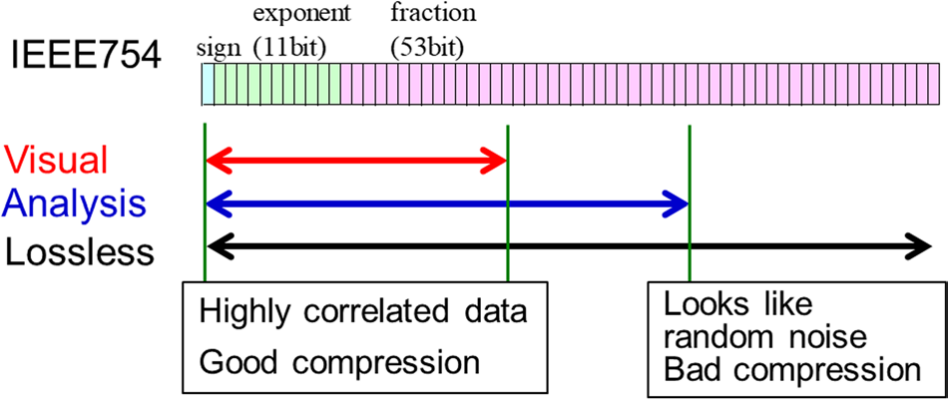
Required number of bits for visualization and analysis.

The required number of bits in the IEEE 754 format differs for different purposes such as visualization and analysis of scientific data, as shown in Fig. 2. Thus, the required number of bits needs to be properly evaluated for each purpose and simulation target. In scientific simulations using the laws of physics, the first fraction bits are correlated in space and time. However, the tailing fraction bits do not always contribute to visualization and analyses and may instead exhibit random noise-like behavior, which negatively affects data compression^27–29,49,50^. A higher compression ratio using only the first fraction bits can be observed if the tailing fraction bits can be neglected. Regarding compression efficiency, both data size and ease of use should be considered. For the latter, a simple solution should not change the Application Programming Interface (API). Thus, the conventional binary format with Huffman coding (ex. gzip), and HDF5 can be used as the data API. A combination of zero padding and data compression (such as Huffman coding) can be effective because the size of information in the zero padded bits becomes negligibly small after Huffman coding.

**Fig. 3:**
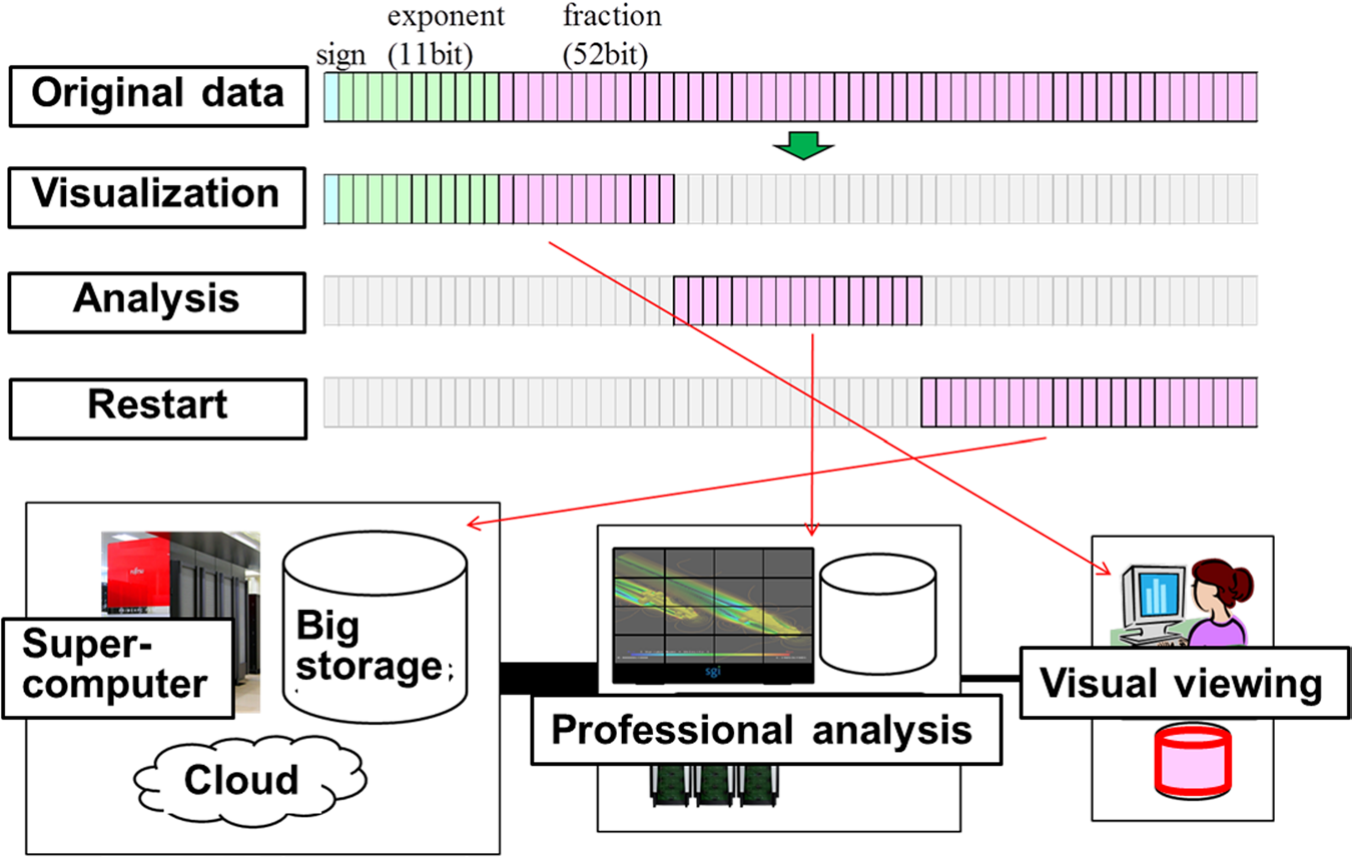
Example application of separated binary files created within the JHPCN-DF. In this example, the required number of bits was 24 bits and 41 bits for visualization and analysis, respectively. 64 bits of double-precision data were split into three 64-bit recordings: [24 bits + 0-padding (40 bits)], [0-padding (24 bits) + 17 bits + 0-padding (23 bits)], and [0-padding (41 bits) + 23 bits]. Huffman cording reduced the total size of the original 64 bits to less than 64 bits.

In our implementation^29,49,50^, the required bit length of each floating-point data was checked for user-specified error levels, such as 0.000001. For the case of IEEE 754 double-precision floating-point data, the stored value of the original variable requires zero padding and a 64-bit integer to record the separated bits necessary to reconstruct higher precision data and the original data (lossless). The recordings in the separated binary files using the JHPCN-DF framework are presented in Fig. 3. In this example, 64 bits of double-precision data were split into three parts: [24 bits + 0-padding (40 bits)], [0-padding (24 bits) + 17 bits + 0-padding (23 bits)], and [0-padding (41 bits) + 23 bits]. Before Huffman coding, the total size of the original 64 bits was 192 bits in memory. After Huffman coding, the total size of the original 64 bits became less than 64 bits. For decoding, the OR-operation for the separated data reconstructs original (lossless) data and/or higher precision data. For the example shown in Fig. 3, lossless data can be obtained using the OR-operation for three 64-bit data recordings: OR([24 bits + 0-padding (40 bits)], [0-padding (24 bits) + 17 bits + 0-padding (23 bits)], and [0-padding (41 bits) + 23 bits]).

## Data Records

The dataset^59^ consists of 150 systems of ring-linear polymer blends, as shown in Table 1. The datasets are available via the Figshare repository.

### Dataset 1

Each filename contains information on the type of ring complex: *N*_ring_, *M*_ring_, *N*_linear_, *M*_linear_, and *f*_ring_. For example, “TwoB_NR120x240_NL20x28800_fr005-D-jhpcndf000001” indicates that the complex was bonded to two ring polymers (as shown in Fig. 3(b)), *N*_ring_ = 120, *M*_ring_ = 240, *N*_linear_ = 20, *M*_linear_ = 28,800, and *f*_ring_ = 0.05. The types of ring complex are indicated by “One,” “TwoB,” “ThreeB,” “TwoC,” and “ThreeC,” which correspond to Fig. 3(a–e), respectively. Note that “D-jhpcndf000001” indicates double-precision binary with JHPCN-DF compression and an error level of 0.00001.

Each file contains the following data:

- Size of PBC box (3 × 8 bytes)
- Positions of beads (3 × *N*_total_ × 8 bytes)
  Here, *N*_total_ = *N*_ring_ *M*_ring_ + *N*_linear_ *M*_linear_. Moreover, 3 × *N*_ring_ × *M*_ring_ × 8 bytes in the second line indicates the positions of the ring polymers. The remaining data indicate the positions of linear chains. In this database, we assumed that the bead order represents the bond connection. *N*_ring_ beads made a single ring polymer, whereas *N*_linear_ beads made a linear chain.
  In addition, the tailing fraction bits of bead positions were also provided with int64 binary; these are indicated with “D-jhpcndf000001XOR” to denote JHPCN-DF compression and the tailing (XOR) parts. Here, the tailing fraction bits were obtained from the XOR-operation between the original data and the double-precision binary with JHPCN-DF compression.
- Tailing fraction bits of positions of beads (3 × *N*_total_ × 8 bytes)

## Technical Validation

### Evaluation of segmented recording data

For the double-precision data generated in the MD simulations, we applied JHPCN-DF compression with user-specified error levels of 0.00001, 0.000001, and 0.0000001. For tests of single-precision binary data, single-precision data were obtained by casting from double-precision data. For single-precision binary analysis, we examined cases with user-specified error levels of 0.1, 0.01, 0.001, and 0.0001. Here, 0.0001 was smaller than the limit from the value range, as mentioned below.

Tables 2 and 3 present the size [bytes] and compression ratio of compressed files for single and double-precision binary recording. Here, we employed three methods to achieve the specified error level of the compressed files: (1) “tar” and “gzip −9” for the segmented recording binary file based on JHPCN-DF, (2) “tar” for the “sz”-compressed file of the lossless binary file, and (3) “tar” for the “sz”-compressed file of the segmented recording binary file with JHPCN-DF. Here, we used version 2.1.8.3 of SZ with the Zstd best compression mode^36^. In the process of generating the compressed files, we monitored the maximum and minimum values of positions: Max = 1981.244394305023 and Min = −1806.817917672729. It should be noted that these values may be inaccurate with single precision. In the case of single precision, from this range and fraction part of 23-bits, as (Max − Min)/2^23^﻿ was approximately 0.00045, the error level cannot be maintained even for a single-precision binary without JHPCN-DF. According to the obtained compression ratios, the results for all compression methods were similar. For all cases, the combination of JHPCN-DF and the SZ-compressor showed the best performance. It should be noted that the increased size of SZ-compressed files for single-precision data with a specified error level of 0.0001 may be a result of insufficient detail parameter tuning. Further analysis of this hypothesis is beyond the scope of this paper.

**Table 2:** Single-precision binary recording: compressed file size [bytes] and confusion matrix of topology judgments.

Specified error level	Compressed size [bytes]	Compression ratio	Confusion matrix	Error ratio
Lossless	1,058,081,560	—	—	0 (ground truth)
0.1	568,736,963 480,696,320 480,542,720	53.75% 45.43% 45.41%	[[180,543, 6,745],[6,864, 1,374,717,848]]	9.898e-06
0.01	697,258,978 679,249,920 668,119,040	65.89% 64.42% 63.14%	[[186,525, 763],[726, 1,374,723,986]]	1.083e-06
0.001	828,550,734 1,056,235,520 805,140,480	78.31% 99.83% 76.09%	[[187,216, 72],[83, 1,374,724,629]]	1.127e-07
0.0001	985,218,102 1,058,918,400 978,309,120	93.12% 100.08% 92.46%	[[187,283, 5],[10, 1,374,724,702]]	1.091e-08

Regarding the compressed size and compression ratio, the upper, middle, and lower records correspond to the results of JHPCN-DF, SZ compression, and both JHPCN-DF and SZ compression, respectively.

**Table 3:** Double-precision binary recording: compressed file size [bytes] and confusion matrix of topology judgments.

Specified error level	Compressed size [bytes]	Compression ratio	Confusion matrix	Error ratio
Lossless	1,460,836,817	—	—	0 (ground truth)
0.00001	1,133,326,989 1,147,484,160 1,111,470,080	77.58% 78.55% 76.08%	[[187,290, 0],[0, 1,374,724,710]]	0
0.000001	1,219,899,212 1,213,665,280 1,187,983,360	83.51% 83.08% 81.32%	[[187,290, 0],[0, 1,374,724,710]]	0
0.0000001	1,265,078,931 1,256,069,120 1,238,814,720	86.60% 85.98% 84.80%	[[187,290, 0],[0, 1,374,724,710]]	0
Single precision	1,058,081,560	72.43%	[[187,289, 1],[0, 1,374,724,710]]	7.273 e-10

Regarding the compressed size and compression ratio, the upper, middle, and lower records correspond to the results of JHPCN-DF, SZ compression, and both JHPCN-DF and SZ compression, respectively.

### Topology analyses using segmented recording data

As a test for the segmented recording data, we evaluated the GLN for topology judgment regarding penetration of a linear chain into a ring polymer using the method proposed by the authors^13,14^. This is because the topology is not conserved if the numerical accuracy is poor. The ratio of correct answers of the topology judgment was used as the evaluation index, which was obtained for several user-specified accuracies. Tables 2 and 3 present the confusion matrix and error ratio of the topology judgment for all pairs of ring polymers and linear chains in all systems. Here, the confusion matrix has been effectively employed as a two-class classification problem in machine learning and is given as [[True Positive (TP), False Negative (FN)], [False Positive (FP), True Negative (TN)]], where “Positive” means that the linear chain penetrated into the ring polymer and “True” means that the topology was preserved between lossless compression and the specified error level. The error ratio was defined as (FP + FN)/(TP + FP + FN + TN).

According to the single-precision binary recording in Table 2, increasing the error level (tolerance) increases misjudgment of the topology. This test provides a good example of the relationship between numerical precision and topology judgment errors. However, regarding the original purpose of achieving recording with topology conservation, the single-precision binary format was insufficient. Moreover, the double-precision data in Table 3 exhibited no error in topology judgment with an error level of 0.00001, whereas the single-precision data exhibited two errors. Consequently, we used the JHPCN-DF method with an error level of 0.00001 to develop the publicly available database of well-equilibrated initial configurations of ring-linear polymer blends.

We also investigated the influence of the size of linear chains (*N*_linear_) because an incorrect judgment is more likely for shorter linear chains due to the limitation of the topology judgment algorithm between a ring polymer and a linear chain^13^. Tables 4 and 5 present the *N*_linear_ dependence of the error ratio of topology judgments. If the error ratio can be optimized for this problem, compression with an error level corresponding to *N*_linear_ is justified.

**Table 4:** Single-precision binary recording: *N*_linear_-dependence of the error ratio of topology judgments.

Specified error level	*N*_linear_ = 10	*N*_linear_ = 20	*N*_linear_ = 40	*N*_linear_ = 80	*N*_linear_ = 160
0.1	1.020e-05	9.678e-06	9.475e-06	9.210e-06	9.898e-06
0.01	1.099e-06	1.096e-06	1.026e-06	1.003e-06	1.105e-06
0.001	1.113e-07	1.240e-07	1.296e-07	6.764e-08	6.764e-08
0.0001	9.864e-09	1.409e-08	1.127e-08	1.127e-08	0

**Table 5:** Double-precision binary recording: *N*_linear_-dependence of the error ratio of topology judgments.

Specified error level	*N*_linear_ = 10	*N*_linear_ = 20	*N*_linear_ = 40	*N*_linear_ = 80	*N*_linear_ = 160
0.00001	0	0	0	0	0
0.000001	0	0	0	0	0
0.0000001	0	0	0	0	0
Single precision	0	0	5.637e-09	0	0

## Supplementary information

Supporting Information

## Data Availability

To decode the JHPCN-DF compression, no special attention was required. (The easy-to-use sample code for generating the LAMMPS input data file is attached in the Supporting Information) To encode/segment the data into two parts with JHPCN-DF compression, as shown for the above data, the main part of the reference code^41^ is as follows: union fi64{ double f; uint64_t i64; }; double fval0,fval1,allowerr,logallo; int i,ntotal,ival,ival2,sval; union fi64 fival,fival1; double *posi_before_compress, *posi_after_compress; uint64_t *tailing_fraction_bits_posi; allowerr = 0.00001 logallo = log(allowerr)/log(2.0); for(i = 0;i < 3*ntotal;i++){ fval0 = posi_before_compress[i]; frexp(fval0,&ival); ival2 = (int)(-logallo + ival); sval = (int)(53-ival2); if(sval > 52) sval = 53; do { sval–; fival.f = fval0; fival.i64 = (fival.i64 ≫ sval); fival.i64 = (fival.i64 ≪ sval); fval1 = fival.f; } while ((fval1-fval0)*(fval1-fval0) >allowerr*allowerr); posi_after_compress[i] = fval1; fival1.f = fval1; fival.f = fval0; tailing_fraction_bits_posi[i] = (fival1.i64 ^ fival.i64); } For software developers, RIKEN has released the open library “JHPCN-DF” at the following GitHub repository: https://github.com/avr-aics-riken/JHPCN-DF.
