# Supplementary material for: Efficient compressed database of equilibrated configurations of ring-linear polymer blends for MD simulations
Source: Sci Data. 2022 Feb 8;9:40. doi: 10.1038/s41597-022-01138-3 (PMC8825841; doi:10.1038/s41597-022-01138-3)
Supplement: Supplementary file 1 — Supporting Information [file 41597_2022_1138_MOESM1_ESM.pdf]

Supporting Information

Efficient Compressed Database of Equilibrated Configurations of Ring-Linear Polymer Blends for MD Simulations

Katsumi Hagita[1], Takahiro Murashima[2], Masao Ogino[3], Manabu Omiya[4], Kenji Ono[5], Tetsuo Deguchi[6], Hiroshi Jinnai[7], Toshihiro Kawakatsu[2]

1. Department of Applied Physics, National Defense Academy, 1-10-20, Hashirimizu, Yokosuka, 239-8686, Japan
2. Department of Physics, Tohoku University, 6-3, Aramaki-aza-Aoba, Aoba-ku, Sendai, 980-8578, Japan
3. Faculty of Informatics, Daido University, 10-3 Takiharu-cho, Minami-ku, Nagoya 457-8530, Japan
4. Information Initiative Center, Hokkaido University, Kita 11, Nishi 5, Kita-ku, Sapporo 060-0811, Japan
5. Research Institute for Information Technology, Kyushu University, 744 Motooka, Nishi-ku, Fukuoka, 819-0395, Japan
6. Department of Physics, Ochanomizu University, 2-1-1 Ohtsuka, Bunkyo-ku, Tokyo 112–8610, Japan
7. Institute of Multidisciplinary for Advanced Materials, Tohoku University, 2-1-1 Katahira, Aoba-ku, Sendai, 980-8577, Japan

corresponding author(s): Katsumi Hagita (hagita@nda.ac.jp)

Table of Contents

A. Instruction to generate the LAMMPS input data from the distributed data
B. Instruction of OVITO to check the generated LAMMPS input data
C. Instruction to reconstruct the lossless data

A. Instruction to generate the LAMMPS input data from the distributed data

To use the distributed data in the MD simulations using LAMMPS [1], we provided the easy-to-use sample code “Bin2LMPdata.c” in the figshare record. As mentioned in the main text, no special attention was required to decode the JHPCN-DF compression. The distributed data can be treated as a standard binary with a commodity C code.

As an example, we provided the instruction for "TwoB_NR120x240_NL20x28800_fr005-D-jhpcndf000001".

(1) In the code “Bin2LMPdata.c”, we should specify the filename (as “fname1”) and the parameters (as “nring”, “mring”, “nlinear”, and “mlinear”).
(2) The code “Bin2LMPdata.c” can be compiled by a standard GNU C compiler (gcc) and/or Intel C compiler (icc).
(3) After a running of the executable binary (a.out), we obtained the LAMMPS input data file named as "TwoB_NR120x240_NL20x28800_fr005-D-jhpcndf000001.data".

B. Instruction of OVITO to check the generated LAMMPS input data.

As an example of visualization of the generated data, we provided the instruction of the visualization software OVITO [2].

(1) Run OVITO binary.

(2) Load file. Select the menu-bar "[File]-[Load file]" and open the LAMMPS input data file. The unwrapped plot is obtained. The tab "Add modification" is often used in the later.

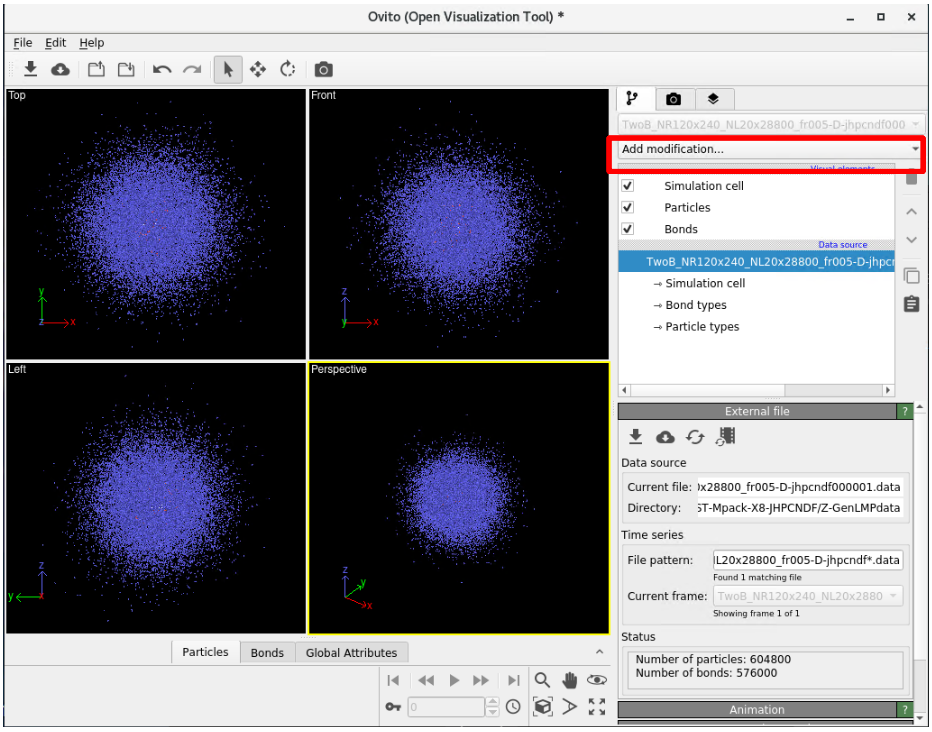

(3) To wrap with the periodic boundary conditions, we use "Wrap at periodic boundaries" in "Add modification". The wrapped plot is obtained. By using the buttons "Focus on the yellow marked window" and "Auto resize", the resized plot is obtained.

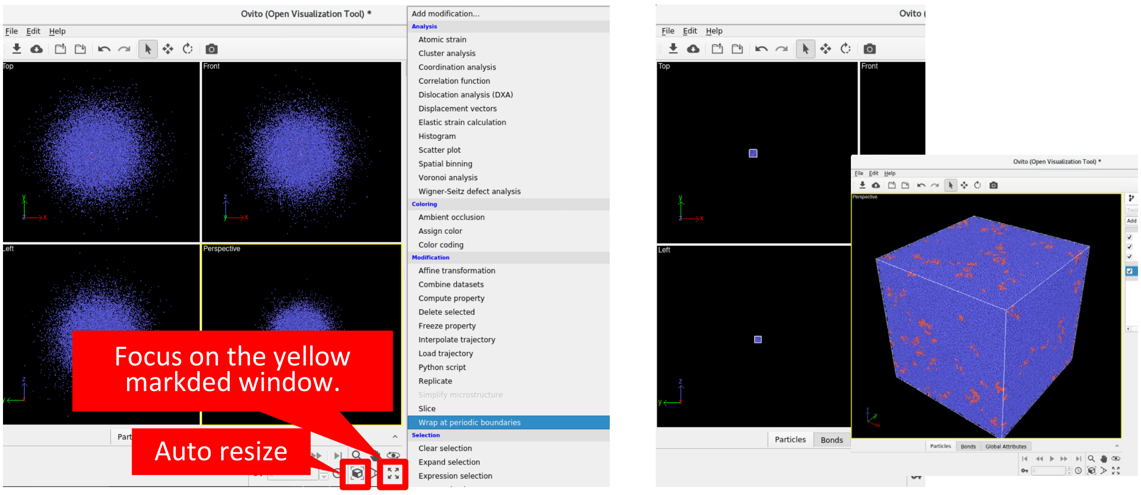

(4) To pickup a single ring, we apply “Expression selection” and “Delete selected” in “Add modification”. The order of the selected “Modifications” is as follows.

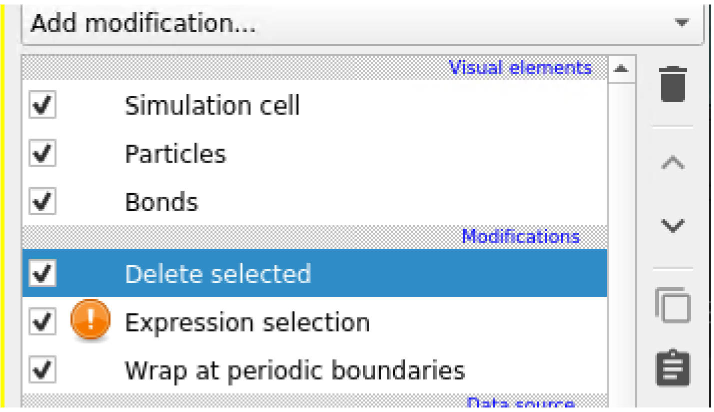

(5) To specify a single ring, we input “ParticleIdentifier < 601 || ParticleIdentifier > 720” as “Boolean expression” for the Modification “Expression selection”. The single plot is obtained.

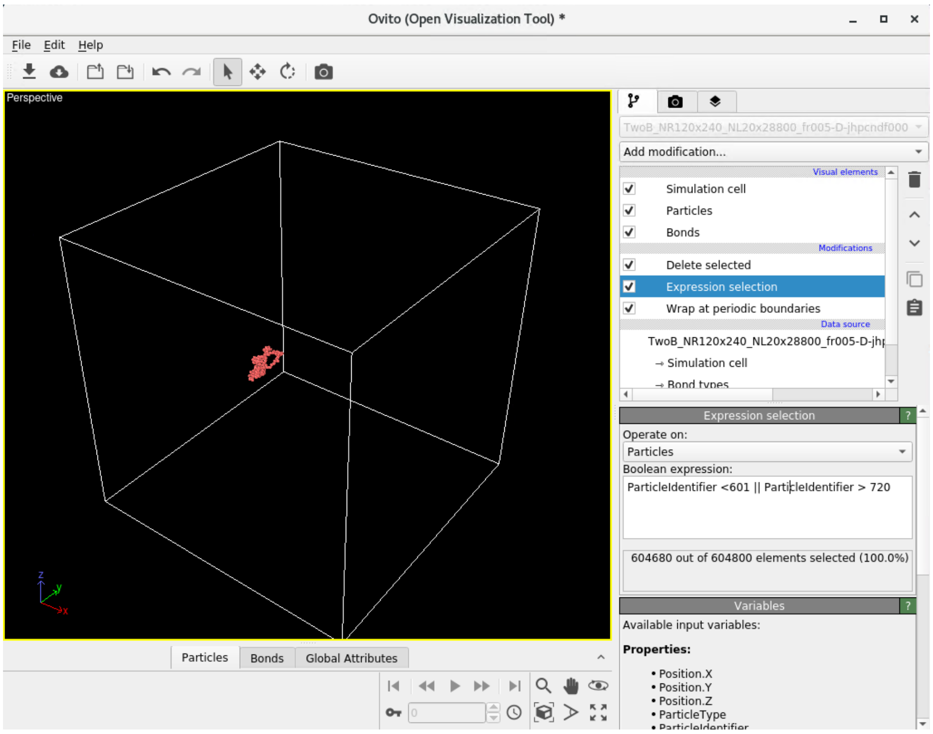

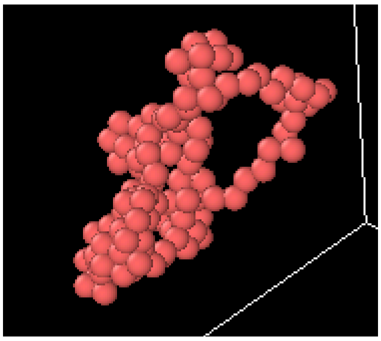

C. Instruction to reconstruct the lossless data

To reconstruct the lossless data from the data "TwoB_NR120x240_NL20x28800_fr005-D-jhpcndf000001" and "TwoB_NR120x240_NL20x28800_fr005-D-jhpcndf000001XOR", we provided the sample code "ReconstLossLess.c" in the figshare record.

(1) In the code "ReconstLossLess.c", we should specify the filenames (as "fname1" and "fname2", and "fname3") and the parameters (as "nring", "mring", "nlinear", and "mlinear").
(2) The code "ReconstLossLess.c" can be compiled by a standard GNU C compiler (gcc) and/or Intel C compiler (icc).
(3) After a running of the executable binary (a.out), we obtained the lossless binary with the same binary format as "TwoB_NR120x240_NL20x28800_fr005-D-jhpcndf000001".

To confirm the lossless data, we can use "Bin2LMPdata.c" as a text-decoder. The original *x*-coordinate of the first particle for the data of "TwoB_NR120x240_NL20x28800_fr005-D" is 89.153661347656. The correspond data of the "printf with %d" statement in "Bin2LMPdata.c" for "TwoB_NR120x240_NL20x28800_fr005-D-jhpcndf000001" is 89.153656. For the reconstructed lossless data from "TwoB_NR120x240_NL20x28800_fr005-D-jhpcndf000001" and "TwoB_NR120x240_NL20x28800_fr005-D-jhpcndf000001XOR", the correspond data is 89.153661.

References

1. Plimpton, S. Fast Parallel Algorithms for Short-Range Molecular Dynamics, *J. Comput. Phys.* 117, 1–19 (1995).
2. Stukowski, A. Visualization and analysis of atomistic simulation data with OVITO – the Open Visualization Tool. *Modelling Simul. Mater. Sci. Eng.* **18**, 015012 (2010).
